# Supplementary material for: Household transmission of SARS-CoV-2 in five US jurisdictions: Comparison of Delta and Omicron variants
Source: PLoS One. 2025 Jan 9;20(1):e0313680. doi: 10.1371/journal.pone.0313680 (PMC11717262; doi:10.1371/journal.pone.0313680)
Supplement: S1 Table — Abbreviations: Ref, reference group; RR, relative risk; CI, confidence interval, a Final model shown in Table 2. b Data shown are the exponentiated interaction terms (ratio of Delta:Omicron relative risks). An interaction term was included in the final model if the term was significant at the α = 0.05 level (i.e. the 95% confidence interval did not include the null). For multilevel variables, an interaction term was included if it was significant at the α = 0.05 level for any level of the variable. c Vaccination status was defined as of the index case date and was categorized as unvaccinated (never received a COVID-19 vaccine), partially vaccinated (completed part of a COVID-19 vaccine series or completed the primary series <14 days prior), fully vaccinated (completed the primary series ≥14 days prior or received a booster dose <7 days prior), or boosted (received an additional vaccine dose beyond the primary series ≥7 days prior). d Number of prior immunologic experiences combined previous infection and vaccination doses to estimate the number of times a person’s immune system had been exposed to SARS-CoV-2 or the vaccine. The possible range of values was zero to four exposures and could be any combination of infection and vaccination. Those with unknown previous infection status were considered to have no previous infection for this composite variable. (DOCX) [file pone.0313680.s001.docx]

**Supplemental Table 1. Models and interaction terms assessed to estimate the adjusted relative risk describing SARS-CoV-2 transmission among household contacts controlling for SARS-CoV-2 variant, index case characteristics and household contact characteristics.**

| **Characteristic** | **Initial model**  **RR (95% CI)** | **Interaction Model 1**  **RR (95% CI)** | **Interaction Model 2**  **RR (95% CI)** | **Interaction Model 3**  **RR (95% CI)** | **Interaction Model 4**  **RR (95% CI)** | **Interaction Model 5^a^**  **RR (95% CI)** | **Interaction Model 6**  **RR (95% CI)** |
| --- | --- | --- | --- | --- | --- | --- | --- |
| **SARS-CoV-2 variant in the household** |  |  |  |  |  |  |  |
| Delta | 1.0 (ref) | 1.0 (ref) | 1.0 (ref) | 1.0 (ref) | 1.0 (ref) | 1.0 (ref) | 1.0 (ref) |
| Omicron | 1.2 (1.0-1.3) | 1.0 (0.7-1.3) | 1.1 (0.5-2.3) | 1.1 (0.9-1.3) | 1.3 (0.8-1.9) | 1.1 (0.9-1.3) | 1.1 (0.9-1.3) |
| **Index case characteristics** | |  |  |  |  |  |  |
| Age group (years) |  |  |  |  |  |  |  |
| 0-4 | 1.0 (ref) | 1.0 (ref) | 1.0 (ref) | 1.0 (ref) | 1.0 (ref) | 1.0 (ref) | 1.0 (ref) |
| 5-11 | 0.8 (0.6-1.0) | 0.7 (0.6-0.9) | 0.8 (0.6-1.0) | 0.8 (0.6-1.0) | 0.8 (0.6-1.0) | 0.8 (0.6-1.1) | 0.8 (0.6-1.0) |
| 12-17 | 0.7 (0.5-0.9) | 0.4 (0.3-0.7) | 0.7 (0.5-0.9) | 0.6 (0.5-0.9) | 0.6 (0.5-0.9) | 0.7 (0.5-0.9) | 0.6 (0.5-0.9) |
| 18-64 | 0.8 (0.7-1.0) | 0.7 (0.6-0.9) | 0.8 (0.7-1.0) | 0.8 (0.6-1.0) | 0.8 (0.6-1.0) | 0.8 (0.7-1.0) | 0.8 (0.6-1.0) |
| ≥65 | 0.9 (0.7-1.2) | 0.8 (0.5-1.1) | 0.9 (0.7-1.2) | 0.9 (0.7-1.2) | 0.9 (0.7-1.2) | 0.9 (0.7-1.2) | 0.9 (0.7-1.2) |
| Symptom status |  |  |  |  |  |  |  |
| Asymptomatic | 1.0 (ref) | 1.0 (ref) | 1.0 (ref) | 1.0 (ref) | 1.0 (ref) | 1.0 (ref) | 1.0 (ref) |
| Symptomatic | 2.0 (1.4-2.9) | 2.0 (1.4-3.0) | 1.9 (1.1-3.5) | 2.0 (1.4-2.9) | 2.0 (1.4-2.9) | 2.0 (1.4-2.9) | 2.0 (1.4-2.9) |
| Vaccination status^b^ | |  |  |  |  |  |  |
| Unvaccinated/partially vaccinated | 1.0 (ref) | 1.0 (ref) | 1.0 (ref) | 1.0 (ref) | 1.0 (ref) | 1.0 (ref) | 1.0 (ref) |
| Fully vaccinated | 0.9 (0.8-1.1) | 0.9 (0.8-1.1) | 0.9 (0.8-1.1) | 0.9 (0.7-1.1) | 0.9 (0.8-1.1) | 0.9 (0.8-1.1) | 0.9 (0.8-1.1) |
| Boosted | 0.9 (0.7-1.1) | 0.8 (0.6-1.1) | 0.9 (0.7-1.1) | 0.8 (0.4-1.8) | 0.8 (0.7-1.1) | 0.8 (0.6-1.1) | 0.8 (0.6-1.1) |
| **Household contact characteristics** | |  |  |  |  |  |  |
| Age group (years) |  |  |  |  |  |  |  |
| 0-4 | 1.0 (ref) | 1.0 (ref) | 1.0 (ref) | 1.0 (ref) | 1.0 (ref) | 1.0 (ref) | 1.0 (ref) |
| 5-11 | 1.3 (1.0-1.7) | 1.3 (1.0-1.7) | 1.3 (1.0-1.7) | 1.3 (1.0-1.7) | 1.5 (1.0-2.2) | 1.3 (1.0-1.7) | 1.3 (1.0-1.7) |
| 12-17 | 1.0 (0.8-1.3) | 1.0 (0.8-1.3) | 1.0 (0.8-1.3) | 1.0 (0.8-1.3) | 0.9 (0.6-1.6) | 1.0 (0.8-1.3) | 1.0 (0.8-1.3) |
| 18-64 | 1.4 (1.1-1.8) | 1.4 (1.1-1.8) | 1.4 (1.1-1.8) | 1.4 (1.1-1.8) | 1.4 (1.0-2.2) | 1.4 (1.1-1.8) | 1.4 (1.1-1.8) |
| ≥65 | 1.5 (1.1-2.2) | 1.5 (1.1-2.2) | 1.5 (1.1-2.2) | 1.5 (1.1-2.2) | 1.4 (0.9-2.4) | 1.5 (1.1-2.2) | 1.5 (1.1-2.2) |
| Time since last vaccine dose (months) | |  |  |  |  |  |  |
| Unvaccinated/partially vaccinated | 1.0 (ref) | 1.0 (ref) | 1.0 (ref) | 1.0 (ref) | 1.0 (ref) | 1.0 (ref) | 1.0 (ref) |
| 0-3 | 1.1 (0.8-1.6) | 1.1 (0.8-1.6) | 1.1 (0.8-1.6) | 1.1 (0.8-1.6) | 1.1 (0.8-1.6) | 0.8 (0.5-1.2) | 1.1 (0.8-1.6) |
| 4-7 | 1.2 (0.9-1.7) | 1.2 (0.9-1.7) | 1.2 (0.9-1.7) | 1.2 (0.9-1.7) | 1.2 (0.9-1.6) | 1.3 (0.9-1.8) | 1.2 (0.9-1.7) |
| ≥8 | 1.2 (0.8-1.6) | 1.2 (0.8-1.6) | 1.2 (0.8-1.6) | 1.2 (0.8-1.6) | 1.1 (0.8-1.6) | 1.2 (0.7-1.8) | 1.2 (0.8-1.6) |
| Number of prior immunologic experiences^c^ |  |  |  |  |  |  |  |
| 0 | 1.0 (ref) | 1.0 (ref) | 1.0 (ref) | 1.0 (ref) | 1.0 (ref) | 1.0 (ref) | 1.0 (ref) |
| 1 | 0.9 (0.7-1.2) | 0.9 (0.7-1.2) | 0.9 (0.7-1.2) | 0.9 (0.7-1.2) | 0.9 (0.7-1.3) | 1.0 (0.7-1.3) | 0.8 (0.5-1.3) |
| 2 | 0.9 (0.6-1.3) | 0.9 (0.6-1.2) | 0.9 (0.6-1.3) | 0.9 (0.6-1.3) | 0.9 (0.7-1.3) | 0.9 (0.6-1.3) | 0.8 (0.6-1.2) |
| 3 | 0.7 (0.5-1.0) | 0.7 (0.5-1.0) | 0.7 (0.5-1.0) | 0.7 (0.5-1.0) | 0.7 (0.5-1.1) | 0.7 (0.4-1.0) | 0.5 (0.3-0.9) |
| **Variant-characteristic interaction terms^b^** | |  |  |  |  |  |  |
| Index case age group (years) |  |  |  |  |  |  |  |
| 0-4 | NA | 1.0 (ref) | NA | NA | NA | NA | NA |
| 5-11 | NA | 1.1 (0.7-1.8) | NA | NA | NA | NA | NA |
| 12-17 | NA | 1.9 (1.0-3.6) | NA | NA | NA | NA | NA |
| 18-64 | NA | 1.2 (0.9-1.6) | NA | NA | NA | NA | NA |
| ≥65 | NA | 1.3 (0.8-2.1) | NA | NA | NA | NA | NA |
| Index case symptom status | |  |  |  |  |  |  |
| Asymptomatic | NA | NA | 1.0 (ref) | NA | NA | NA | NA |
| Symptomatic | NA | NA | 1.1 (0.5-2.3) | NA | NA | NA | NA |
| Index case vaccination status^c^ | |  |  |  |  |  |  |
| Unvaccinated/partially vaccinated | NA | NA | NA | 1.0 (ref) | NA | NA | NA |
| Fully vaccinated | NA | NA | NA | 1.1 (0.8-1.4) | NA | NA | NA |
| Boosted | NA | NA | NA | 1.0 (0.5-2.3) | NA | NA | NA |
| Household contact age group (years) | |  |  |  |  |  |  |
| 0-4 | NA | NA | NA | NA | 1.0 (ref) | NA | NA |
| 5-11 | NA | NA | NA | NA | 0.8 (0.5-1.3) | NA | NA |
| 12-17 | NA | NA | NA | NA | 1.1 (0.6-2.0) | NA | NA |
| 18-64 | NA | NA | NA | NA | 0.9 (0.6-1.5) | NA | NA |
| ≥65 | NA | NA | NA | NA | 1.1 (0.6-2.0) | NA | NA |
| Household contact time since last vaccine dose months) | |  |  |  |  |  |  |
| Unvaccinated/partially vaccinated | NA | NA | NA | NA | NA | 1.0 (ref) | NA |
| 0-3 | NA | NA | NA | NA | NA | 1.7 (1.2-2.6) | NA |
| 4-7 | NA | NA | NA | NA | NA | 0.9 (0.7-1.2) | NA |
| ≥8 | NA | NA | NA | NA | NA | 1.0 (0.7-1.5) | NA |
| Household contact number of prior immunologic experiences^d^ | |  |  |  |  |  |  |
| 0 | NA | NA | NA | NA | NA | NA | 1.0 (ref) |
| 1 | NA | NA | NA | NA | NA | NA | 1.3 (0.8-2.1) |
| 2 | NA | NA | NA | NA | NA | NA | 1.1 (0.9-1.4) |
| 3 | NA | NA | NA | NA | NA | NA | 1.5 (0.9-2.6) |

Abbreviations: Ref, reference group; RR, relative risk; CI, confidence interval

^a^ Final model shown in Table 2.

^b^ Data shown are the exponentiated interaction terms (ratio of Delta:Omicron relative risks). An interaction term was included in the final model if the term was significant at the α = 0.05 level (i.e. the 95% confidence interval did not include the null). For multilevel variables, an interaction term was included if it was significant at the α = 0.05 level for any level of the variable.

^c^ Vaccination status was defined as of the index case date and was categorized as unvaccinated (never received a COVID-19 vaccine), partially vaccinated (completed part of a COVID-19 vaccine series or completed the primary series <14 days prior), fully vaccinated (completed the primary series ≥14 days prior or received a booster dose <7 days prior), or boosted (received an additional vaccine dose beyond the primary series ≥7 days prior).

^d^ Number of prior immunologic experiences combined previous infection and vaccination doses to estimate the number of times a person’s immune system had been exposed to SARS-CoV-2 or the vaccine. The possible range of values was zero to four exposures and could be any combination of infection and vaccination. Those with unknown previous infection status were considered to have no previous infection for this composite variable.
